# Composition-Tunable Optical Properties of Zn_*x*_Cd_(1 − *x*)_S Quantum Dot–Carboxymethylcellulose Conjugates: Towards One-Pot Green Synthesis of Multifunctional Nanoplatforms for Biomedical and Environmental Applications

**DOI:** 10.1186/s11671-017-2212-8

**Published:** 2017-07-05

**Authors:** Alexandra A. P. Mansur, Herman S. Mansur, Anderson J. Caires, Rafael L. Mansur, Luiz C. Oliveira

**Affiliations:** 1 Center of Nanoscience, Nanotechnology and Innovation - CeNano(2)I, Department of Metallurgical and Materials Engineering, Universidade Federal de Minas Gerais—UFMG, Belo Horizonte, Brazil; 2 Department of Chemistry, Universidade Federal de Minas Gerais—UFMG, Belo Horizonte, Brazil; 3 Federal University of Minas Gerais, Av. Antônio Carlos, 6627 – Escola de Engenharia, Bloco 2 – Sala 2233, Belo Horizonte, MG 31.270-901 Brazil

**Keywords:** Semiconductor quantum dot nanoparticles, Nanomaterials, Semiconductor-biopolymer interfaces, Nanophotocatalyst, Core-shell nanostructures

## Abstract

Quantum dots (QDs) are colloidal semiconductor nanocrystals with unique properties that can be engineered by controlling the nanoparticle size and chemical composition by doping and alloying strategies. However, due to their potential toxicity, augmenting their biocompatibility is yet a challenge for expanding to several biomedical and environmentally friendly applications. Thus, the main goal of this study was to develop composition-tunable and biocompatible Zn_*x*_Cd_1 − *x*_S QDs using carboxymethylcellulose polysaccharide as direct capping ligand via green colloidal aqueous route at neutral pH and at room temperature for potential biomedical and environmental applications. The ternary alloyed QDs were extensively characterized using UV–vis spectroscopy, photoluminescence spectroscopy (PL), transmission electron microscopy (TEM), X-ray diffraction (XRD), electron energy loss spectroscopy (EELS), and X-ray photoelectrons spectroscopy (XPS). The results indicated that Zn_*x*_Cd_(1 − *x*)_S QDs were surface stabilized by carboxymethylcellulose biopolymer with spherical morphology for all composition of alloys and narrow sizes distributions ranging from 4 to 5 nm. The XRD results indicated that monophasic ternary alloyed Zn_*x*_Cd_1 − *x*_S nanocrystals were produced with homogenous composition of the core as evidenced by EELS and XPS analyses. In addition, the absorption and emission optical properties of Zn_*x*_Cd_1 − *x*_S QDs were red shifted with increasing the amount of Cd^2+^ in the alloyed nanocrystals, which have also increased the quantum yield compared to pure CdS and ZnS nanoparticles. These properties of alloyed nanomaterials were interpreted based on empirical model of Vegard’s law and chemical bond model (CBM). As a proof of concept, these alloyed-QD conjugates were tested for biomedical and environmental applications. The results demonstrated that they were non-toxic and effective fluorophores for bioimaging live HEK293T cells (human embryonic kidney cells) using confocal laser scanning fluorescence microscopy. Moreover, these conjugates presented photocatalytic activity for photodegradation of methylene blue used as model organic industrial pollutant in water. Hence, composition-tunable optical properties of ternary Zn_*x*_Cd_1 − *x*_S (*x* = 0–1) fluorescent alloyed QDs was achieved using a facile eco-friendly aqueous processing route, which can offer promising alternatives for developing innovative nanomaterials for applications in nanomedicine and environmental science and technology.

## Background

Traditionally, alloys have been used for several centuries to create new materials with improved mechanical, structural, thermal, electrical properties leading to the development of high-performance materials, which cannot be achieved with each component separately. However, only very recently the interest in nanoalloys (also referred to as alloy nanoclusters or alloy nanoparticles) arises, as they constitute a new type of advanced nanoscale materials, which can have unique properties very distinct from those of individual atoms and molecules or original bulk matter [1–4]. One of the major reasons for interest in alloy nanoparticles is the fact that their chemical and physical properties may be tuned by varying the composition and atomic ordering as well as the dimension of the clusters [4, 5]. Besides the metal-based nanomaterial alloys, nano-sized semiconductor nanocrystals (referred to as quantum dots, QDs) have increasingly called the attention of the scientists, researchers, and manufacturers because of their broad range of potential applications in electronics, optics, magnetic, sensors, biosensors, and biomedical fields [6–11]. In that sense, quantum dots based on metal chalcogenide alloys, mostly of group II–VI semiconductors (type MX, M = Cd, Zn, Pb; X = Te, Se, S), are currently under intensive study in many research fields such as in optoelectronics, high-density memory, quantum-dot lasers, and lately for biosensing and biolabeling because they exhibit tunable optical properties (i.e., bandgap energy structure) by adjusting the chemical composition and size [5, 7, 12–15]. Since the bandgap engineering of ternary and quaternary alloyed QDs can be achieved via controlling their composition (relative constituent stoichiometry) in addition to their sizes and internal structures, it is therefore feasible to design and tune their optical properties, which are not readily viable to binary QDs [16–19]. This can be achieved by creating a solid solution (i.e., an alloy) of two semiconductors with different energy gaps, where an increase in the bandgap energy is generally observed with increasing the concentration of the wider bandgap semiconductor, either with cation (i.e., metal constituent) or anion (i.e., chalcogenide constituent) alloyed QDs [20, 21]. Nonetheless, the development of QDs based on Zn–Cd–S alloys using one-pot “greener” aqueous processes with biocompatible ligands are narrowly reported in the literature, where the large majority of studies report the production of QDs at high temperatures by organometallic routes, microwave-assisted synthesis, and using toxic organic solvents [15, 17, 18, 21–25]. High-temperature decomposition methods based on trioctylphosphine oxide (TOPO), commonly used as capping ligand for improved quantum yields, results in hydrophobic QDs insoluble in aqueous medium. However, biological and medical applications require QDs that are water-soluble at physiological conditions, biocompatible, and functionalized with biomolecules for targeting purposes. Hence, the development of novel strategies of surface functionalization and bioconjugation remains an important challenge. Yet, no report was found in the consulted literature addressing the synthesis of Zn_*x*_Cd_1 − *x*_S QDs functionalized with biopolymer ligands based on cellulose derivatives for multiple potential purposes, including cell bioimaging and photocatalytic activity for the degradation of organic dye pollutants.

Thus, herein, we report the synthesis and comprehensive characterization of novel ternary alloyed Zn_*x*_Cd_1 − *x*_S QDs (*x* = 0 → 1) with composition-tunable optical properties using carboxymethylcellulose as a biocompatible and eco-friendly capping ligand produced directly by means of a single-step green colloidal process in aqueous media at room temperature. They proved to be suitable nanoplatforms for live cell bioimaging or heterogeneous photocatalysis of methylene blue organic dye.

## Methods

### Materials

All of the reagents and precursors, including zinc chloride (Sigma-Aldrich, USA, ≥98%, ZnCl_2_), cadmium perchlorate hydrate (Aldrich, USA, Cd(ClO_4_)_2_·6H_2_O), and sodium sulfide hydrate (Synth, Brazil, >98%, Na_2_S·9H_2_O) were used as received. Carboxymethylcellulose sodium salt (CMCel, Fluka Chemical, USA), degree of substitution (DS) 0.84 and medium viscosity (870 mPa s, 2% in H_2_O) was used as capping ligand. Deionized water (DI water, Millipore Simplicity^TM^) with a resistivity of 18 MΩ cm was used to prepare the solutions and the procedures were performed at room temperature (RT, 23 ± 2 °C), unless specified otherwise.

### Synthesis of Quantum Dot Conjugates—Zn_*x*_Cd_1 − *x*_S/CMCel

CMCel solution (1% *w*/*v*) was prepared by adding sodium carboxymethylcellulose powder (0.5 g) to a 50 mL of water and stirring at room temperature until complete solubilization occurred. For controlling the molar ratios of cations, premixed Zn^2+^ and Cd^2+^ solutions were prepared from the individual stock solutions (ZnCl_2_ and Cd(ClO_4_)_2_·6H_2_O) at 1 × 10^−3^ mol L^−1^ total cation concentration (M) but with Zn^2+^:Cd^2+^ molar ratios of 100:0 (*x* = 1.0), 75:25 (*x* = 0.75), 50:50 (*x* = 0.50), 25:75 (*x* = 0.25), and 0:100 (*x* = 0).

Zn_*x*_Cd_1 − *x*_S nanoparticles were synthesized via an aqueous route at room temperature as follows: 2 mL of CMCel solution and 45 mL of deionized water were added to a flask. The pH was measured and it was close to neutral (pH∼7.0). Under magnetic stirring, 20.0 mL of the metal precursor solution (1 × 10^−3^ mol L^−1^) at the Zn^2+^:Cd^2+^ different molar ratios and 4.0 mL of the sulfide source solution (Na_2_S·9H_2_O, 1.0 × 10^−2^ mol L^−1^) were added to the flask (the S^2−^:M^2+^ molar ratio was kept at 2:1, i.e., excess of sulfide) and stirred for 10 min. The QDs colloidal dispersions produced were stable and homogeneous. ZnS dispersion was colorless while CdS dispersion was light yellow. The dispersions of ternary Zn_*x*_Cd_1 − *x*_S nanoparticles exhibit colors between clear and light yellow with continuous color gradient as a function of the Zn^2+^:Cd^2+^ molar ratio.

These colloidal dispersions were concentrated and purified using an Amicon® Ultra Filter (Millipore) with a 100,000 molecular mass (M_W_) cut-off cellulose membrane. Centrifugation was conducted and, after the first cycle, the QDs were washed 4 times with DI water. Centrifugal forces caused the removal of excess reagents through the membrane into a filtrate vial. After purification, the samples were stored at RT until further use.

### Physicochemical Characterization of Quantum Dot Conjugates

Ultraviolet–visible (UV–vis) spectroscopy measurements were performed using Perkin-Elmer, Inc. (USA) equipment (Lambda EZ-210) in transmission mode with samples in a quartz cuvette over a wavelength range between 600 and 190 nm. All of the experiments were conducted in triplicate (*n* = 3) unless specifically noted, and the data were presented as the mean ± standard deviation.

The photoluminescence spectroscopy (PL) of the Zn_*x*_Cd_1 − *x*_S/CMCel conjugates was performed based on spectra acquired at RT using a violet diode laser module at *λ*_exc_ = 405 nm (150-mW, Roithner LaserTechnik, Germany) coupled to a USB4000 VIS-NIR spectrophotometer (Ocean Optics, Inc., USA). All of the tests were performed using a minimum of four repetitions (*n* ≥ 4). Quantum yield (QY) was measured according to the procedure using Rhodamine 6G (Sigma, USA) in ethanol as the standard at *λ*_excitation_ = 405 nm [26]. Nanostructural characterization of the QDs was based on the images and electron diffraction patterns (ED) using Tecnai G2-20-FEI (FEI Company, USA) transmission electron microscope (TEM) at an accelerating voltage of 200 kV. In all of the TEM analyses, the samples were prepared by placing a drop of a dilute QD suspension onto carbon-coated copper grids (Electron Microscopy Sciences, USA) and allowing them to dry at room temperature overnight. The QD size and size-distribution data were obtained based on the TEM images by measuring at least 150 randomly selected nanoparticles using image processing program (ImageJ, version 1.50, public domain, National Institutes of Health) [27].

X-ray diffraction (XRD) patterns were recorded using PANalytical (UK) Empyrean diffractometer (Cu–Kα radiation with *λ* = 1.5406 Å). Measurements were performed in the 2*θ* range from 3° to 70° with steps of 0.06°. For the sample preparation, concentrated colloidal QD dispersions were dropped onto glass slides and oven dried at 40 ± 1 °C for 12 h.

Energy-filtered transmission electron microscopy (EFTEM) with low-loss electron energy-loss spectroscopy (EELS) was performed by using Tecnai G20 TEM operating at 200 kV accelerating voltage and equipped with GATAN GIF energy imaging filter. The cadmium composition map was obtained using the Cd M edge at 404 eV, and the Zn composition map was obtained using the Zn L edge at 1020 eV, using similar sample preparation procedure of TEM analysis.

X-ray photoelectron spectroscopy (XPS) analysis was performed using Mg–Kα as the excitation source (Amicus spectrometer, Shimadzu, Japan). All peak positions were corrected based on C 1s binding energy (284.6 eV). For sample preparation, concentrated QD colloidal medium was dropped onto glass slides and dried in a vacuum desiccator at RT for 48 h.

Dynamic light scattering (DLS) and zeta potential (ZP) analyses were performed using ZetaPlus instrument (Brookhaven Instruments Corporation, 35-mW red diode laser light, wavelength *λ* = 660 nm) with a minimum of ten replicates. The ZP measurements were performed at 25.0 °C ± 2 °C under the Smoluchowski approximation method. For the DLS measurements, the colloidal solutions of QDs were filtered three times through a 0.45-μm aqueous syringe filter (Millex LCR 25 mm, Millipore).

### Biological Characterization of QD Conjugates

#### Evaluation of Cytotoxicity by MTT Cell Viability Assay

MTT (3-(4,5-dimethylthiazol-2yl) 2,5-diphenyl tetrazolium bromide) experiments were performed to evaluate the toxicity of QDs dispersions. MTT assays were conducted according to ISO 10993-5:2009 (Biological evaluation of medical devices: Tests for in vitro cytotoxicity) using kidney cell line of a human embryonic culture (HEK293T). HEK293T cells were kindly provided by Prof. M.F Leite (Department of Physiology and Biophysics, UFMG), and they were cultured in DMEM with 10% FBS, penicillin G sodium (10 units mL^−1^), streptomycin sulfate (10 mg mL^−1^), and amphotericin-b (0.025 mg mL^−1^), all from Gibco BRL (NY, USA), in a humidified atmosphere of 5% CO_2_ at 37 °C.

HEK293T cells on passage 68 were plated (3 × 10^4^ cells/well) in 96-well plates. Cell populations were synchronized in serum-free media for 24 h. After that, the media volume was suctioned and replaced with DMEM media containing 10% FBS for 24 h. The samples of CdS, ZnS, and Zn_0.50_Cd_0.50_S QDs colloidal solutions were added to individual wells at final concentrations of 10 nM. For MTT assay, control samples were designed as follows: control group (cell culture with DMEM medium); positive control (1.0% Triton X-100, Sigma-Aldrich, USA); and negative control (chips of sterile polypropylene Eppendorf, 1 mg mL^−1^, Eppendorf, Germany). After 120 h, all media were aspirated and replaced with 60 μL of culture media containing serum to each well. MTT (5 mg mL^−1^, Sigma-Aldrich, USA) was added to each well and incubated for 4 h in an oven at 37 °C and 5% CO_2_. Next, 40 μL SDS (Sigma-Aldrich, USA) solution/4% HCl was placed in each well and incubated for 16 h in an oven at 37 °C and 5% CO_2_. Then, 100 μL was removed from each well and transferred to a 96-well plate. The absorbance was measured at 595 nm on iMark™ Microplate Absorbance Reader (Bio-Rad) with a 595-nm filter. Percentage cell viability was calculated according to Eq. 1. The values of the controls (wells with cells and no samples) were set to 100% cell viability.

1 $$ \mathrm{Cell}\ \mathrm{viability} = \left(\mathrm{Absorbance}\ \mathrm{of}\ \mathrm{sample}\ \mathrm{and}\ \mathrm{cells}\right)/\left(\mathrm{Absorbance}\ \mathrm{of}\ \mathrm{control}\right) \times 100\ \% $$

#### Cellular Uptake of QD Conjugates by Laser Scanning Confocal Microscopy

The evaluation of the QD conjugates as fluorescent biological probes was performed using confocal laser scanning microscopy after exposing ZnS and Zn_0.50_Cd_0.50_S QDs to HEK293T cells. For the cellular internalization evaluation, HEK293T cells on passage 68 were plated (5 × 10^4^ cells per well) in 24-well plate. The cells were incubated for 4 days in 5% CO_2_ at 37 °C and synchronized for 24 h, and the ZnS and Zn_0.50_Cd_0.50_S colloidal suspensions with the medium solution (DMEM) at final concentration of 500 nM were added to the cells. Next, the cells were incubated in 5% CO_2_ at 37 °C for 1 h and washed with phosphate buffered saline (PBS, Gibco, Brazil). In the sequence, the cells were fixed with paraformaldehyde (4%) for 30 min, washed three times with PBS, and cover slips were mounted with Hydromount (Fisher Scientific Ltd., UK) for posterior analysis in confocal laser scanning fluorescence microscopy (Zeiss LSM Meta 510, Carl Zeiss; excitation 488 nm argon laser; emission filter 505–530 nm). For the control, cells were incubated with only in the original medium with 10% FBS (autofluorescence).

### Photocatalytic Activity of QD Conjugates for Environmental Applications

The photocatalytic activities of the ZnS and Zn_0.50_Cd_0.50_ conjugates were evaluated via the photocatalytic degradation of molecule methylene blue (MB) as model organic pollutant molecule under ultraviolet (UV) light irradiation.

The experiments were performed at room temperature using 15 mL of a solution of MB (20 mg L^−1^) containing 0.1 mL of each conjugate catalyst under mechanic stirring (330 rpm) and a mercury lamp radiation source (15 W, at *λ* = 254 nm). At certain time intervals, an aliquot of the material was collected and analyzed by UV–vis spectroscopy (Shimadzu-UV-1601 PC) to detect the dye concentration based on Beer–Lambert curves established to correlate the absorbance with the dye concentration at the characteristic maximum absorption wavelength (*λ* = 664 nm). The degradation efficiency was calculated using Eq. 2 where “*C*_o_” is the initial concentration of the dye (*t* = 0) and “*C*” is the concentration of MB at a given time of radiation exposure (*t*). A control experiment was performed without the presence of the catalytic conjugates.

2 $$ \mathrm{Degradation}\ \mathrm{efficiency}\ \left(\%\right) = \left[\left({C}_0- C\right)/{\mathrm{C}}_0\right] \times 100\ \% $$

## Results and Discussion

### Physicochemical Characterization of Quantum Dot Conjugates

**Fig. 1:**
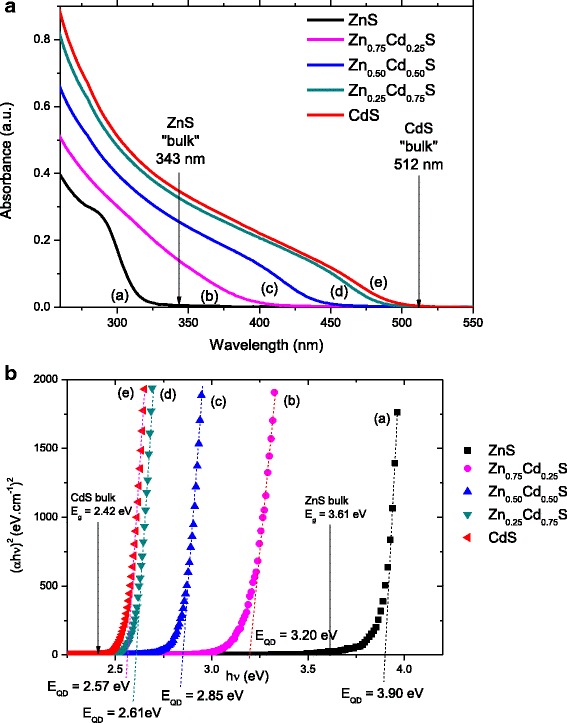
UV–visible and optical absorption spectroscopy of conjugates. **a** UV–vis absorption spectra and **b** optical absorption spectra (relation) for ZnS *(a)*, Zn_0.75_Cd_0.25_S *(b)*, Zn_0.50_Cd_0.50_S *(c)*, Zn_0.25_Cd_0.75_S *(d)*, and CdS *(e)*

Figure 1A shows de UV–vis spectra for Zn_*x*_Cd_1 − *x*_S (*x* = 1.0, 0.75, 0.50, 0.25, and 0) series. ZnS nanoparticles (Fig. 1A(a)) presents a sharp absorption edge at approximately 290 nm associated with the first excitonic transition indicating nucleation and growth of ZnS (*x* = 1) nanoparticles, blue shifted from the bulk value at 343 nm [28] due to the “quantum confinement regime.” Analogously, CdS (*x* = 0) colloidal suspension (Fig. 1A(e)) presented a broad absorption band from 430 to 470 nm, blue shifted when compared with the original bulk value (*λ* = 512 nm, [28]) indicating the formation of CdS QDs. The optical absorption spectra of Zn_*x*_Cd_1 − *x*_S ternary systems show a systematic red shift associated with the changes in the absorption onset of the S_0_ → S_1_ transitions as the Cd content increases which originates from the wider bandgap ZnS (*E*_g_ = 3.61 eV) relative to the narrower bandgap CdS (*E*_g_ = 2.42 eV) [28].

Bandgap values for the prepared metal sulfide QDs (*E*_QD_) at different proportions of [Zn,Cd]:S were extracted from the UV–vis absorbance curves using the “TAUC relation” (Fig. 1B). The estimated *E*_QD_ values are 3.90, 3.20, 2.85, 2.61, and 2.57 eV for ZnS, Zn_0.75_Cd_0.25_S, Zn_0.50_Cd_0.50_S, Zn_0.25_Cd_0.75_S, and CdS, respectively. Therefore, the tunable bandgap effect was indeed verified, as the excitonic transitions for the Zn_*x*_Cd_1 − *x*_S ternary solid solutions (Fig. 1A (b–d)) continuously red shifted (i.e., shifted to lower energy) with increasing the amount of Cd^2+^, which demonstrated the formation of Zn_*x*_Cd_1 − *x*_S nanoalloys.

**Fig. 2:**
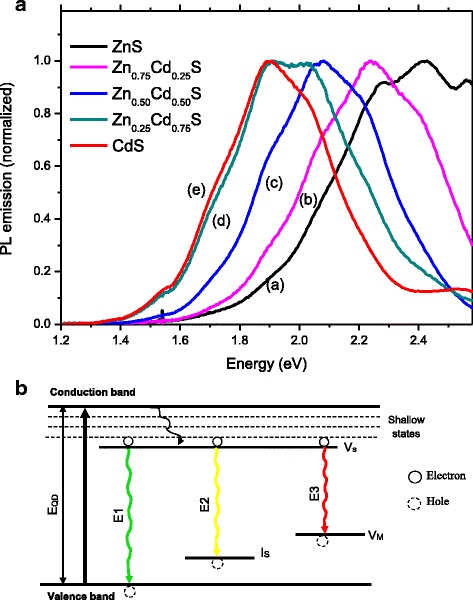
Photoluminescence spectroscopy of conjugates. **a** PL emission spectra for Zn_*x*_Cd_1 − *x*_S (*x* = 1.0 *(a)*, 0.75 *(b)*, 0.50 *(c)*, 0.25 *(d)*, and 0 *(e)*. **b** The energy band structure suggested for three main emissions of alloyed Zn_*x*_Cd_1 − *x*_S quantum dots

Typical room-temperature PL spectra of the Zn_*x*_Cd_1 − *x*_S nanoparticles stabilized by CMCel are presented in Fig. 2A. The Zn_*x*_Cd_1 − *x*_S QDs showed multi-colored light emissions arising predominantly from defect activated photoluminescence. That means, lattice point defects (vacancies, V, and interstitials atoms, I) act as efficient traps for electrons, holes, and exciton charge carriers, leading to radiative recombination at energies lower than the band-to-band optical transition (i.e., offering non-radiative pathways) [29, 30]. As previously reported [31, 32], due to the small size of QDs and the intrinsic lattice point defects introduced by the synthesis at room temperature and aqueous routes, band edge (excitonic) luminescence was not observed and the QYs were typically ≤2.0% (1.0% for ZnS; 1.8% for Zn_0.50_Cd_0.50_S; 0.2% for CdS).

The occurrence, population, and depths of the traps determine the pathway that the electron–hole pairs (i.e., exciton, e^−^/h^+^) generated by irradiation will follow during emission. Considering the synthesis process developed in this work, using the stoichiometric molar ratio of M^2+^:S^2−^ = 1:2 (i.e., higher sulfide to cation ratio), the occurrence of vacancies of metals (V_M_: V_Cd_ or V_Zn_) and sulfur at interstitial sites at lattice (I_S_) are expected. Moreover, the strong interaction between the metallic cations and the anionic carboxylate species from CMCel, which are fully deprotonated at the pH of synthesis (pK_a_ = 4.8, R–COOH → R–COO^−^) can cause the formation of vacancies of sulfur (Vs) at the QD surface. Thus, considering the combination of the types of point defects (V_M_, V_Cd_, V_Zn_, and I_S_) and the similarity of the emission curves for Zn_*x*_Cd_1 − *x*_S, three main radiative emissions were observed in the nanoparticles, which were assigned to the energy band diagram depicted in Fig. 2B and summarized in Table 1, supported by the literature [33–37].

**Table 1:** Summary of types and energies of Zn_*x*_Cd_1 − *x*_S QD emissions

Emission		ZnS	Zn_0.75_Cd_0.25_S	Zn_0.50_Cd_0.50_S	Zn_0.25_Cd_0.75_S	CdS
Excitonic emission						
Defect activated emission (eV)	E1 (V_S_–V_B_)	2.56	2.42	2.21	2.02	2.03
	E2 (V_S_–I_S_)	*2.42 green*	*2.24 green*	*2.07 yellow*	*1.91 red*	*1.90 red*
	E3 (V_S_–V_M_)	2.27	2.07	1.89	1.71	1.71

Italicized values indicate the maximum PL intensity and visible emission color

First, there is a non-radiative pathway from valence band and energy level of V_S_ due to shallow trap states. In the sequence, the emission E1 can be associated with transitions involving sulfur vacancies (V_S_) and valence band (V_B_). Emission E2 can be assigned to the recombination of trapped electrons at Vs and holes trapped at interstitial metal (I_S_) point defects and E3 is attributed to the recombination between vacancy trap states (V_S_–V_M_). The minor emission bands at higher wavelengths can be assigned to surface defects [33, 35]. Despite the nature of the emission, peaks in the PL spectra shifted to lower energy with the increase of Cd molar ratio in the QDs demonstrated the composition-tunable emission effect of nanoalloys. These values of red shift observed in the PL spectroscopy support the results of previous sections, and they are further strong evidence of the formation of alloyed nanocrystals.

**Fig. 3:**
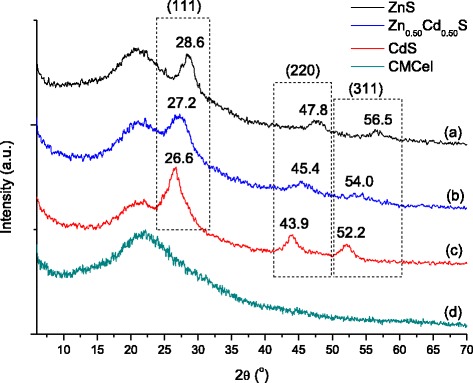
X-Ray diffraction analysis of conjugates. XRD patterns of ZnS *(a)*, Zn_0.50_Cd_0.50_S *(b)*, CdS *(c)*, and CMCel ligand *(d)*

The diffraction peaks observed in the XRD pattern of ZnS QDs (Fig. 3a) at 2*θ* at 28.6 ± 0.1°, 47.8 ± 0.1°, and 56.5 ± 0.1° can be assigned to the planes (111), (220), and (311) of ZnS cubic lattice structure (Zinc blend also referred to as sphalerite, JCPDS 05-0566). Analogously, the peaks at 2*θ* values of 26.6 ± 0.1°, 43.9 ± 0.1°, and 52.2 ± 0.1° (Fig. 3c) can be indexed to the planes (111), (220), and (311) of the cubic structure CdS (ICCD 89-0440), respectively. The Zn_0.50_Cd_0.50_S sample (Fig. 3b) also has a cubic crystal phase with diffraction peaks shifted relative to the two binary systems and located at an intermediary value between ZnS and CdS. As the ionic radius of Zn^2+^ is smaller than that of Cd^2+^, the addition of Cd atoms to ZnS structure caused diffraction peaks to shift to lower angles with a correspondent increase of the unit–cell volume of the Zn_0.50_Cd_0.50_S sample in comparison with ZnS. Thus, it evidenced that a homogeneous alloyed solid solution has been achieved by controlling the stoichiometric ratio of Zn:Cd. In addition, the broad band present at 2*θ*∼21.8° (Fig. 3d) is characteristic of the CMCel polymer used for the chemical stabilization of the metal sulfide QDs [38].

**Fig. 4:**
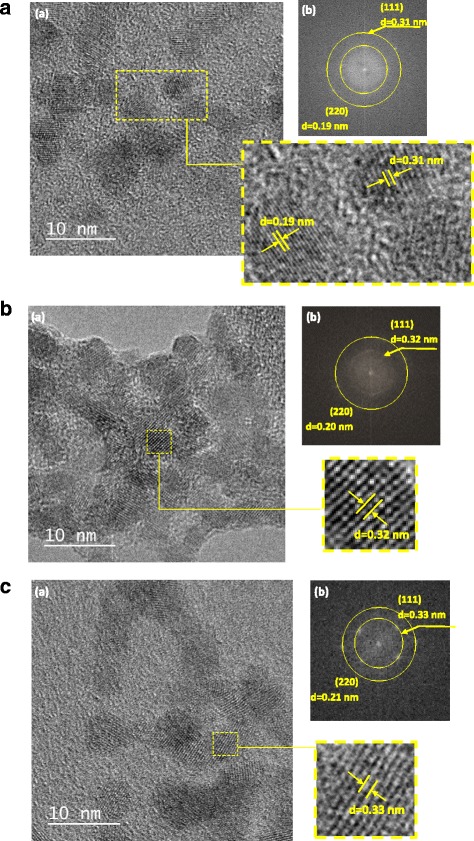
Transmission electron microscopy characterization of conjugates; *(a)* TEM image with SAED patterns (detail) and *(b)* diffraction rings attributed to (111) and (220) planes of the cubic lattice (*d* = interplanar distance) for **a** ZnS, **b** Zn_0.50_Cd_0.50_S, and **c** CdS QDs

In order to access the morphological features and the sizes of the Zn_*x*_Cd_1 − *x*_S systems (*x* = 1.0, 0.50, and 0), TEM analysis was performed and the typical images are showed in Fig. 4A (ZnS), Fig. 4B (Cd_0.50_Zn_0.50_S), and Fig. 4C (CdS). TEM images confirm the formation of QDs with reasonable spherical shape and similar sizes (average diameter ± standard deviation) of 4.9 ± 0.5 nm for CdS, 4.3 ± 0.3 nm for ZnS, and 4.3 ± 0.5 nm for Zn_0.50_Cd_0.50_S. The clear two-dimensional crystal lattice stripes obtained by selected area electron diffraction (SAED patterns) in the TEM images demonstrate the crystalline nature of the QDs and indicate the formation of nanocrystals with cubic structure and lattice parameters consistent with the 2*θ* diffraction angles obtained by XRD.

From the materials science perspective, the properties of the semiconductor nanoalloys produced were interpreted considering empirical and semi-empirical models. Therefore, Vegard’s law empirical rule widely used in metallurgy to predict the physical and optical properties of materials was used to validate the relationship that the lattice constant and bandgap energy would be changed depending on the concentration of the constituents [18, 39, 40]. Therefore, by the combination of wide-bandgap (ZnS, *E*_g_ = 3.61 eV) with medium-bandgap (CdS, *E*_g_ = 2.42 eV) semiconductors [28], it was predicted a linear reduction of the energy of absorption of the alloyed metal sulfide system as the concentration of Cd^2+^ was increased, following the Vegard’s law, Eq. 3.

3 $$ {E_{\mathrm{g}}}^{\mathrm{alloy}}\left(\mathrm{Z}{\mathrm{n}}_x\mathrm{C}{\mathrm{d}}_{1\hbox{-} x}\mathrm{S}\right) = x\ {E}_{\mathrm{g}}\left(\mathrm{Z}\mathrm{nS}\right) + \left(1\ \hbox{-}\ x\right)\ {\mathrm{E}}_{\mathrm{g}}\left(\mathrm{CdS}\right),\ 1.0 < x < 0 $$

**Fig. 5:**
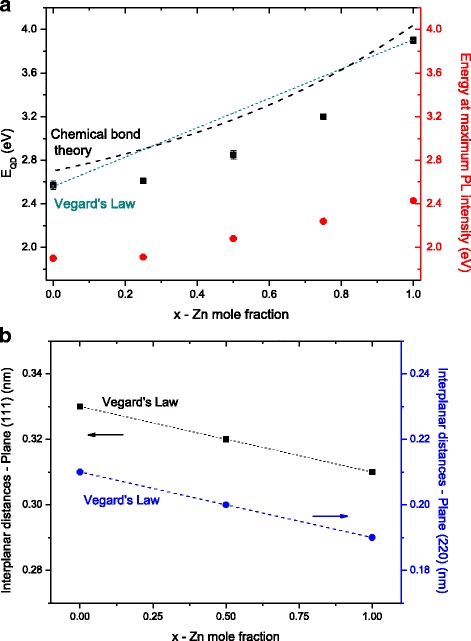
Empirical and semi-empirical models of semiconductor nanoalloys. **a** Non-linear relationship of *E*_QD_ (*squares*) and PL wavelength (*circles*) as a function of the molar content of Zn^2+^. *Dotted line* indicates Vegard’s law (calculated) and *dashed line*, the expected values based on Chemical Bond Theory (from reference [45]). **b** Linear relationship of lattice parameters as a function of the concentration of Zn^2+^ in the material (data from ED analysis)

However, it can be observed in Fig. 5a that the experimental results of bandgap energies and PL emission of the Zn_*x*_Cd_1 − *x*_S ternary nanoalloys presented a deviation to lower values as a general trend from the Vegard’s law plot (dotted line), except for pure QDs (i.e., ZnS or CdS), which were used as reference for constructing this equation. Both optical properties, absorption and emission, were significantly affected by the composition of the Zn_*x*_Cd_1 − *x*_S alloys but with distinct energies from the predicted linear behavior of Vegard’s law. In general, similar to Vegard’s law [39], other models are used for calculating the correlation of optical properties with the size and composition of semiconductor nanocrystals, such as effective mass approximation (EMA) [41], semi-empirical tight-binding model (TBM) [42], and chemical bond model (CBM) [43–45]. Nonetheless, they usually overestimate the bandgap energies under the quantum confinement of the exciton mainly because of simplified assumptions such as by considering a spherical volume of the nanocrystallite, minimizing the complexity and reducing the parameters involved [42, 44]. Although not accurate and valid for all ternary and quaternary alloyed QDs, the chemical bond model (CBM) [43–45], which is not a linear function, is suggested as more adequate for correlating the effect of quantum dependence of optical properties (bandgap energy) with the concentration of alloyed QDs such as Zn_*x*_Cd_1 − *x*_S (dashed line). It can be noted that the CBM plot from the literature [45] with the similar system but with different surface ligand and alloy compositions (Zn_*x*_Cd_1 − *x*_S, *x* = 0 to 0.42, and QD size of 4.4 nm) presented overestimated values of approximately ~200 meV compared to our experimental data. However, the shape of the curve resemble more similarly than linear Vegard’s law, due to the combination of parameters related to quantum confinement (inner QD) and chemical interactions with ligands at the surface (outermost layer of QD) in the CBM model [44, 45].

It is worth mentioning that the red shift of the emission with respect to absorption spectra (known as Stokes shift) increased with raising the Zn^2+^ concentration in the Zn_*x*_Cd_1 − *x*_S, from *x* = 0 to 1.0 (Fig. 5a) due to the complex mechanism of separation and recombination of charges in the nanoalloys. Essentially, based on Brus’ equation [41], who developed the first theoretical calculation for semiconductor nanoparticles named EMA model (Eq. 4), an exciton is considered to be confined to a spherical volume of the nanocrystallite and the mass of electron and hole is replaced with effective masses (*m*_*e*_ and *m*_*h*_) to define the wave function. Hence, the higher reduced mass of exciton (Eq. 5, 1/μ) of ZnS (*m*_e_^*^ *= 0.25, m*_h_^*^ *= 0.59*) compared to CdS (*m*_e_^*^ *= 0.21, m*_h_^*^ = 0.80) increased its relative blue shift by approximately 6% (∆*E*_g_, Eq. 6, where *h* is the Planck’s constant) associated with the quantum confinement, which was indeed verified by increasing the concentration of Zn^2+^ in the alloyed QDs of Zn_*x*_Cd_1 − *x*_S.

4 $$ {E}_{\mathrm{QD}} = {E}_{\mathrm{g}}+\Delta {E}_{\mathrm{g}} $$

5 $$ 1/\mu = 1/{m_e}^{*} + 1/{m_h}^{*} $$

6 $$ \Delta {E}_{\mathrm{g}}\cong {h}^2/8\mu {R}^2 $$

Nonetheless, a distinct behavior was observed for the physical properties (e.g., interplanar distances), where a linear correlation was observed by varying the ternary alloy composition in agreement with the literature [20]. Figure 5b shows the lattice spacing for (111) and (220) planes calculated from ED patterns (TEM images). The interplanar distances exhibit a linear behavior upon changing the *x* values according to Vegard’s law evidencing that the Zn_0.50_Cd_0.50_S system is an alloyed QD instead of a mixture of CdS and ZnS nanocrystals.

**Fig. 6:**
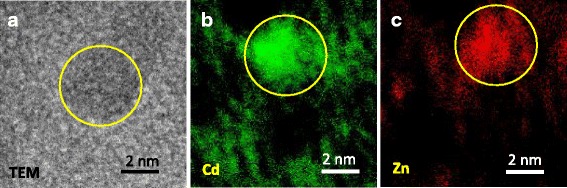
EELS and EFTEM analyses of conjugates. EFTEM Elemental maps of Cd and Zn in samples of Zn_0.50_Cd_0.50_S quantum dots. **a** EFTEM zero energy loss image of an individual Zn_0.50_Cd_0.50_S quantum dot. **b** EELS signals of Cd forming the composition map (Cd M edge at 404 eV). **c** EELS signals of Zn forming the composition map (Zn L edge at 1020 eV)

To perform a more in-depth characterization of Zn_0.50_Cd_0.50_S QDs at nanoscale order, EFTEM coupled with EELS and XPS measurements were performed. Energy-filtered transmission electron microscopy (EFTEM) with low-loss electron energy-loss spectroscopy (EELS) yields new possibilities for the investigation of alloyed ternary nanomaterials [46, 47]. Therefore, the elemental distribution of Zn_0.50_Cd_0.50_S QDs was assessed using EELS/EFTEM techniques to investigate the distribution of Cd and Zn in the synthesized Zn_0.50_Cd_0.50_S QDs, which was selected as a typical alloyed ternary system. Figure 6a shows a zero energy loss image of an isolated QD, and Fig. 6b, c shows the chemical composition maps of Cd and Zn at nanometer resolution. The uniform distribution of the Zn and Cd elements demonstrated the formation of homogeneous alloyed Zn_0.50_Cd_0.50_S QDs, supporting the XRD and TEM results. Moreover, the thermodynamics and kinetics favored the formation of homogenous alloyed ternary QDs as the Gibbs free energy of formation of ZnS and CdS are of the same magnitude (~180–200 kJ mol^−1^) and the solubility product constant are very low (ksp~10^−25^–10^−27^) in the presence of excess of sulfides in the reaction medium at room temperature.

**Fig. 7:**
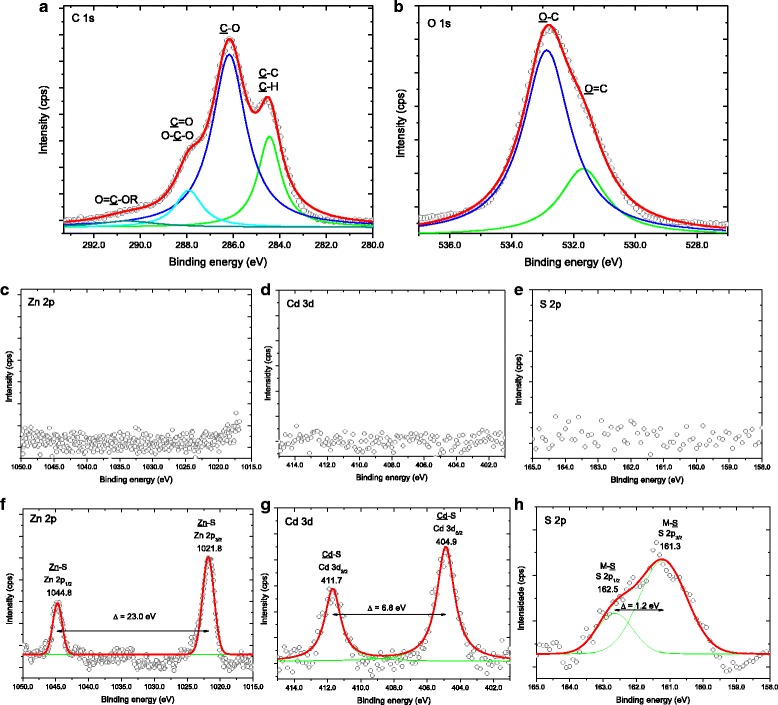
X-ray photoelectron spectroscopy of conjugates. XPS spectra of C 1s (**a**), O 1s (**b**), Zn 2p (**c**), Cd 3d (**d**), and S 2p (**e**) regions at the surface of Zn_0.50_Cd_0.50_S ternary system and XPS spectra of Zn 2p (**f**), Cd 3d (**g**), and S 2p (**h**) regions after etching

Figure 7a–e shows typical XPS spectra obtained directly at the surface of the conjugate composed of Zn_0.50_Cd_0.50_S-CMCel. Figures 7a, b shows representative spectra of C 1s and O 1s regions, respectively. Both spectra are showed several peaks that were identified by deconvolution procedure [48]. C 1s spectrum revealed different chemical bonds of carbon atoms: C–H and C–C bonds at binding energy of 284.5 eV; C–O bond at 286.2 eV; one peak at 287.9 eV assigned to C=O and O–C–O bonds; and O=C–OR bonds at 290.6 eV [48, 49]. Peaks of O=C and O–C at 531.7 and 532.9 eV, respectively, were observed in the O 1s region [49]. These XPS spectra are compatible with the chemical structure of CMCel. In addition, XPS spectra at the surface of the quantum dot–CMCEL conjugates in the regions related to Zn 2p (Fig. 7c), Cd 3d (Fig. 7d), and S 2p (Fig. 7e) indicated no detectable signal from zinc, cadmium, and sulfur, respectively. This result demonstrated the presence of an organic shell of CMCel used as capping agent of QD nanoparticles for controlling the nucleation and limiting the growth of the alloyed nanocrystals just formed in the colloidal media within the quantum confinement regime. Due to the expected repulsion between negatively charged CMC chains associated with carboxylate groups, this organic shell is probably composed of a monolayer surrounding the semiconductor core, which is complex to be precisely measured [50]. Thus, in order to get the distribution of the elements at the surface underneath this CMCel layer, ion bombardment with argon ions (Ar^+^, 1 cycle, 10 s, emission current 10 mA, and beam voltage 0.5 kV) was applied for removing the organic shell. The corresponding elemental composition of the QD surface obtained by XPS spectroscopy taken for Zn 2p, Cd 3d, and S 2p regions is showed in Fig. 7f–h. First, it can be observed that the three chemical elements of the ternary systems were detected, in agreement with the previous results that indicated the formation of ternary nanoalloy. In Fig. 7f, the peaks at 1021.8 and 1044.8 eV correspond to the Zn 2p_3/2_ and Zn 2p_1/2_ levels, respectively, and are associated with the Zn (2p) transitions in ZnS. The spin-orbit components (Zn 2p_3/2_ and Zn 2p_1/2_) are separated by a binding energy interval of approximately 23.0 eV [49, 51–53]. The two strong peaks in Fig. 7g at 411.7 eV (Cd 3d_3/2_) and 404.9 eV (Cd 3d_5/2_) are generally assigned to Cd–S bonding in CdS. The difference between the binding energies of Cd 3d_5/2_ and Cd 3d_3/2_ is 6.8 eV, in agreement with the literature [30, 49, 53]. The S 2p_3/2_ peak (Fig. 7h) and S 2p_1/2_ peaks were found at 161.3 and 162.5 eV, respectively, (Δ = 1.2 eV), which can be assigned to sulfur in metal sulfides (M–S) [49, 51–53]. Elemental analysis data was determined from the integrated area of Cd 3d, Zn 2p, and S 2p signals with appropriate atomic sensitivity factors using the XPS software for data processing (Vision Processing, Kratos).

**Fig. 8:**
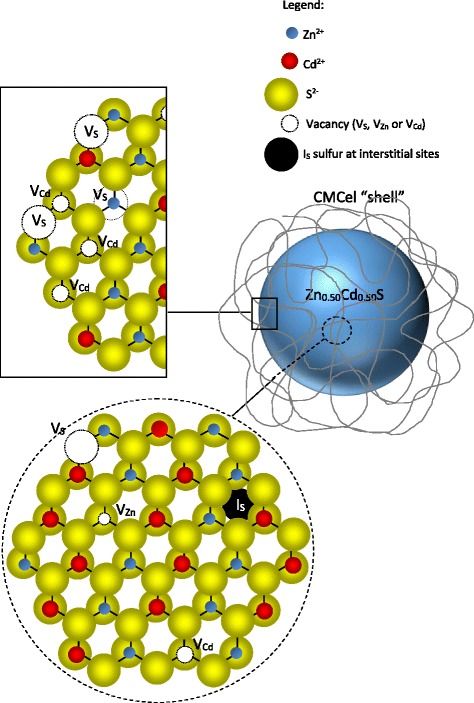
Drawing of Zn_0.50_Cd_0.50_S quantum dot stabilized by CMC polymer; Schematic representation of Zn_0.50_Cd_0.50_S quantum dot (not to scale)

Despite neglecting effects of spherical particle geometry, small QD size, and preferential sputtering, the outmost surface of the QDs presented atomic concentrations (%) of Zn, Cd, and S of 50 ± 4%, 16 ± 2%, and 33 ± 3%, respectively. As expected, distinct from the homogenous composition of the “inner” portion of the nanocrystals, the XPS results of the surface indicated depletion in sulfur content and, therefore, a metal enriched surface (with [Zn^2+^] > [Cd^2+^]). This metal-rich outmost layer can be assigned to the formation of complexes at the surface involving the cations (i.e., Cd^2+^ and Zn^2+^) and groups of the polymer capping ligands [54–57] that compete with sulfide (S^2−^) ions. The relatively higher concentration of Zn^2+^ compared to Cd^2+^ at the QD surface is attributed to the hardness parameter derived from electronegativity, where Zn(II) forms more stable complexes with both oxygen and nitrogen than Cd(II). The stability of complexes of divalent ions in the transition metal series with chelators containing oxygen or nitrogen donors often follows the Irving–Williams order [58, 59]. Conversely, the concentration of sulfides at the surface of QDs is decreased by the repulsion of the anionic carboxylate groups of the CMCel polymer ligand. In addition, this ZnS-rich outermost layer may act as an encapsulating coating of wider bandgap over the homogeneous nanocrystalline “core” that physically separates the surface of the optically active core and reduces the number of dangling bonds that can act as non-radiative surface states that quench PL intensity [60, 61]. Hence, the XPS results evidenced that QD conjugates were produced with core-shell nanostructure composed of alloyed ternary Zn_*x*_Cd_1 − *x*_S inorganic core and carboxymethylcellulose organic shell uniformly dispersed and stabilized in aqueous media as depicted in Fig. 8.

Zeta potential (ζ) measurements evidenced the interactions of QDs with carboxylate groups at the nanoparticle–polymer interfaces and the role of these groups in the stabilization of the QDs. The measured ζ-potentials were all negative values, −52 ± 5 mV (ZnS), −53 ± 6 mV (ZnCdS), and −56 ± 8 mV (CdS), lower than −72 mV of CMCel solution, probably because of metal complexes remained at the nanocrystal–polymer interface (QD–CMCel). In addition, these highly negative values (i.e., ζ ≤ −50 mV) indicated that the nanoparticles were electrostatically stabilized by CMCel carboxylate functional groups avoiding the growth and agglomeration of the nanocrystals, which is crucial for quantum-size confinement effects. Moreover, the morphological and stability features of these colloidal ZnCdS conjugates in aqueous medium were assessed by DLS analysis. Thus, the DLS results showed that the systems were produced with hydrodynamic diameters (H_D_) of 12.5 ± 1.3 nm, 11.8 ± 0.8 nm, and 8.0 ± 0.5 nm for ZnS, ZnCdS, and CdS, respectively. The H_D_ is assigned to the sum of contributions from the inorganic QDs “core” and the CMCel organic “shell” of the conjugates, including the effect of solvation layers and the lateral extension of the capping ligands. The DLS measurements clearly indicated a lower volume of solvation for the CdS that may be associated with the type, extension, and/or stability of the Cd^2+^ chelate complex with chemical groups of the carboxymethylcellulose compared to Zn^2+^, which probably caused the higher contraction of the polymeric shell around the Cd-based QD inorganic core.

### Biological Characterization of Zn_*x*_Cd_1 − *x*_S QD Conjugates

#### Evaluation of Cytotoxicity by MTT Cell Viability Assay

**Fig. 9:**
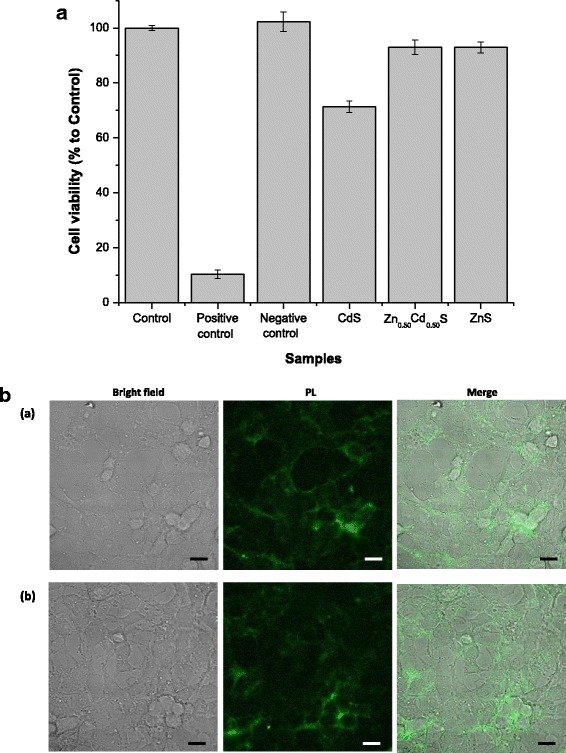
MTT cell viability in vitro assay of conjugates. **a** MTT cell viability assay of HEK293T cells after 120 h of contact of cells with ZnS, Zn_0.50_Cd_0.50_S, and CdS conjugates. **b** Confocal microscopy imaging of the internalization of the *(a)* Zn_0.50_Cd_0.50_S and *(b)* ZnS conjugates in the HEK293T cells (*scale bar* = 10 μm)

MTT (3-[4,5-dimethylthiazol-2-yl]-2,5-diphenyltetrazolium bromide) assay accesses the mitochondrial activity response of cells, which is suitable for evaluating the cell viability and cytotoxicity towards the QD conjugates. The results of MTT assay after 120 h of contact of the cells with ZnS, Zn_0.50_Cd_0.50_S, and CdS conjugates at concentration of 10 nM are presented in Fig. 9A. It demonstrated that, within the statistical variation, no difference was found in the cell viability after 5 days of incubation with the ZnS and Zn_0.50_Cd_0.50_S conjugates when compared to the control condition validating the hypothesis of non-toxicity associated with ZnS and alloyed Zn_0.50_Cd_0.50_S conjugates. Conversely, for CdS samples, as previously reported in the literature [62, 63], an adverse reduction of approximately 30% of cell viability response (~70% viability) was verified, which was attributed predominately to the potential cytotoxicity caused by release of Cd^2+^. Therefore, it was demonstrated that the CMCel polymer played multiple functions for chemically stabilizing and rendering biocompatible ZnS and Zn_0.50_Cd_0.50_S conjugates. Moreover, the Zn-rich outmost surface of Zn_0.50_Cd_0.50_S conjugates, besides enhancing the optical properties compared to pure binary QDs (i.e., ZnS and CdS) as discussed in previous section, it may have also played a relevant role for improving the biocompatibility by hindering the contact of the Cd-alloyed core with the water medium and, therefore, impeding the release of Cd^2+^.

#### Cellular Uptake of Zn_*x*_Cd_1 − *x*_S QD Conjugates by Laser Scanning Confocal Microscopy

In the present study, as a proof of concept, the uptake process of Zn_0.50_Cd_0.50_S and ZnS QD conjugates by live cells was performed by laser scanning confocal microscopy. CdS conjugates were not tested as it has already proved to reduce cell viability by the MTT assay results discussed in the previous section. On the other hand, ZnS QDs have been reported to be safe and non-toxic for in vitro endocytosis and cellular imaging assays. Thus, for the cellular internalization assay, HEK293T cells were incubated for 1 h with Zn_0.50_Cd_0.50_S and ZnS conjugates at final concentration of 500 nM. After this period of incubation, images were captured using laser confocal microscopy for the purpose of visualizing the internalization of the developed surface-biofunctionalized ZnS and Zn_0.50_Cd_0.50_S QDs. Figure 9B shows that the Zn_0.50_Cd_0.50_S (A) and ZnS (B) conjugates were effectively internalized by HEK293T cells. Based on these confocal microscopy images, it was verified that Zn_0.50_Cd_0.50_S and ZnS conjugates were cytocompatible as they penetrated through the cell membrane and were found homogeneously distributed inside the cellular cytoplasm. Therefore, these findings combined with MTT results highlight the suitability of using ZnS and alloyed Zn_0.50_Cd_0.50_S QDs capped by CMCel ligand as fluorescent nanoprobes for in vitro cellular imaging and labeling applications. Although it is a fascinating area of research, a more in-depth investigation is beyond the scope of this work and will certainly be subject of future studies.

### Photocatalytic Activity of Zn_*x*_Cd_1 − *x*_S QD Conjugates for environmental Applications

**Fig. 10:**
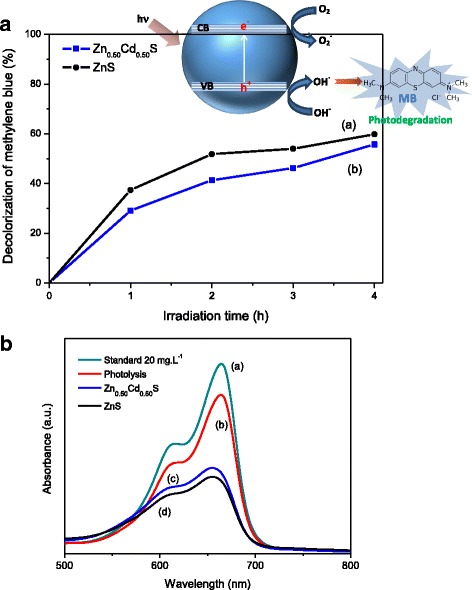
Photocatalysis application of conjugates. **a** Decolorization of methylene blue (MB) in aqueous solution by nanophotocatalyst ZnS *(a)* and Zn_0.50_Cd_0.50_S *(b). Inset drawing*: schematic organic dye degradation by conjugates. **b** Absorption spectra of MB aqueous solution: reference solution *(a)* and degraded by light (control) *(b)*, Zn_0.50_Cd_0.50_S *(c)* and ZnS *(d)* conjugates under UV light after 4 h

Photocatalysis is a rapidly expanding technology for wastewater treatment. For that reason, using a multifunctional approach, here it is presented as a proof of concept, preliminary tests using the ZnS and alloyed Zn_0.50_Cd_0.50_S conjugates as nano-photocatalysts for the degradation of methylene blue (MB) used as a model organic dye pollutant under UV irradiation. The results of degradation efficiency and UV–vis spectra after 4 h of irradiation are summarized in Fig. 10A, B, respectively. Based on Eq. 2, it was found that ZnS conjugates bleached MB relatively faster (first-order rate constant *k* = 0.37 h^−1^) than Zn_0.50_Cd_0.50_S sample (*k* = 0.27 h^−1^) within the first 2 h but with similar degradation efficiency (58% ± 2%) after 4 h of photocatalytic test (Fig. 10A). As both conjugates presented equivalent QD sizes (i.e., 4.3 nm) and surface areas, the minor difference observed for the catalysis kinetics rate can be associated with the presence of a Zn-rich outmost surface layer in the alloyed QD (Zn_0.50_Cd_0.50_S). It is suggested that the wider bandgap surface layer of ZnS over the narrower bandgap Zn_0.50_Cd_0.50_S nanocrystal decreased the density of surface defects (i.e., energy trap states) promoting faster excitonic recombination process inside the semiconductor core. Therefore, this diminution of charge carrier separation (i.e., electron–hole pairs) caused the reduction of generated reactive species crucial for the photocatalytic activity to degrade MB molecules.

Analogously, it can be observed the reduction of absorbance associated with the color of MB at *λ* = 664 nm (Fig. 10B) caused by the photo-oxidation of the MB molecule, shifting the absorption peaks to lower wavelength (blue shift). This photodegradation is related to chemical species generated by electron–hole pairs (QD + hυ → h^+^/e^−^) from irradiated QDs (i.e., ZnS or Zn_0.50_Cd_0.50_S, Fig. 10A, inset drawing). The oxidation mechanism is primarily governed by the valence band holes (h^+^), which are powerful oxidants, and they can react with water or surface-bound chemisorbed hydroxyl groups (OH^−^) producing hydroxyl radicals (OH•). Most organic photodegradation reactions utilize the strong oxidizing power of the holes directly or indirectly produced by excitation of nanomaterials [64–66]. It is reasonable to consider that this degradation of MB may have followed a double-stage process. Initially, the negatively charged CMCel polymer is likely to have attracted MB molecules (i.e., positively charged) leading to the adsorption process, which does not degrade or promote decolorization of MB. In the sequence, the photo-oxidation of the MB dye occurred by the photocatalytic process, which was enhanced by previous adsorption favoring the charge exchange at the conjugate-MB interfaces (i.e., h^+^/e^−^ scavenging).

Regarding to toxicity, some researchers have raised concerns about the environmental safety related to the use of nanomaterials composed with toxic elements such as Cd-containing QDs motivated by the possibility of photo-oxidation with eventual releasing of hazardous elements in the medium [67, 68]. However, despite the hypothetical possibility, it is not likely to occur in these alloyed conjugates because of the biocompatible organic shell encapsulating the inorganic core, the extremely low concentration of the metallic elements (i.e., from μM to nM), associated with the very low water solubility of their compounds (e.g., oxides, sulfates, hydroxides) minimizing eventual risk of contamination.

Thus, these results demonstrated that ZnS and Zn_*x*_Cd_1 − *x*_S semiconductor conjugates were surface functionalized by carboxymethylcellulose using a novel green sustainable process, which can offer promising alternatives as nanoplatforms in environmental and biomedical applications.

## Conclusions

In this study, it is presented the synthesis and characterization of new ternary alloyed Zn_*x*_Cd_1-*x*_S semiconductor QDs using carboxymethylcellulose as a biocompatible polymer capping ligand directly produced via eco-friendly colloidal process in aqueous media and at room temperature. XPS results evidenced the mechanism of stabilization of the Zn_*x*_Cd_1 − *x*_S nanoalloys dominated by the chemical interactions of metal-rich surface (Zn^2+^ > Cd^2+^ > S^2−^) with the carboxylates and hydroxyls groups of the polymer ligands. The EELS, TEM, XRD, and selected area electron diffraction analyses ruled out the formation of core-shell or multiphasic systems, demonstrating that homogenous Zn_*x*_Cd_1 − *x*_S nanoalloys were produced with optical absorption and emission dependent on the concentration of Zn^2+^. In addition, it was observed that the composition of ternary Zn_0.5_Cd_0.5_S alloys improved the luminescence quantum yield compared to the pure binary systems (ZnS and CdS QDs). These optical properties of Zn_*x*_Cd_1 − *x*_S nanoalloys were studied based on empirical model of Vegard’s law and chemical bond model (CBM), where the differences observed of the values were assigned to the bandgap structure of each system. Moreover, in order to provide insights about potential applications of these alloyed-QD conjugates, they were tested for live cell imaging and for photocatalysis of organic molecules. The results demonstrated that ZnS and Zn_0.5_Cd_0.5_S conjugates were non-toxic and behaved as effective fluorophores for in vitro imaging live human embryonic kidney cells (HEK293T) with confocal fluorescence microscopy. Additionally, these alloyed-QD conjugates presented photocatalytic activity for photodegradation of methylene blue, which was used as model organic industrial pollutant in water. Hence, composition-tunable optical properties of ternary Zn_*x*_Cd_1 − *x*_S (*x* = 0–1.0) fluorescent alloyed QDs was verified based on a facile eco-friendly process. It is foreseen that this class of alloyed fluorescent semiconductor nanocrystals offers several possibilities of bandgap engineering for multiple applications such as biolabeling and bioimaging in nanomedicine or as nano-photocatalyst for environmental purposes in water treatment.
